# Temporal Gene Expression Profiling of the Wheat Leaf Rust Pathosystem Using cDNA Microarray Reveals Differences in Compatible and Incompatible Defence Pathways

**DOI:** 10.1155/2007/17542

**Published:** 2007-09-25

**Authors:** Bourlaye Fofana, Travis W. Banks, Brent McCallum, Stephen E. Strelkov, Sylvie Cloutier

**Affiliations:** ^1^Cereal Research Centre, Agriculture and Agri-Food Canada, 195 Dafoe Road, Winnipeg, MB, Canada R3T 2M9; ^2^Department of Agricultural, Food and Nutritional Sciences, University of Alberta, 410 Ag/For Building, Edmonton, AB, Canada T6G 2P5

## Abstract

In this study, we detail the construction of a custom cDNA spotted microarray containing 7728 wheat ESTs and the use of the array to identify host genes that are differentially expressed upon challenges with leaf rust fungal pathogens. Wheat cultivar RL6003 (Thatcher *Lr1*) was inoculated with *Puccinia triticina* virulence phenotypes BBB (incompatible) or TJB (7-2) (compatible) and sampled at four different time points (3, 6, 12, and 24 hours) after inoculation. Transcript expression levels relative to a mock treatment were measured. One hundred ninety two genes were found to have significantly altered expression between the compatible and incompatible reactions. Among those were genes involved in photosynthesis, the production of reactive oxygen species, ubiquitination, signal transduction, as well as in the shikimate/phenylpropanoid pathway. These data indicate that various metabolic pathways are affected, some of which might be used by RL6003 to mount a coordinated defense against an incompatible fungal pathogen.

## 1. INTRODUCTION

Leaf rust, caused by the heteroecious basidiomycete *Puccinia triticina* Eriks, is one of the most important diseases of wheat worldwide. Serious yield losses can result as a consequence of its broad distribution and potential to develop rapidly under optimal environmental conditions. Furthermore, the ability of *P. triticina* to form new races that can attack previously resistant cultivars, along with the capacity of fungal spores to travel long distances, can make full control of leaf rust difficult. Due to its importance, epidemiological [1], genetic [2, 3], and molecular aspects [4, 5] of the disease have been studied extensively. As a consequence, the wheat-*P. triticina* interaction is well defined in genetic terms and stages of infection [4, 6–8]. Scanning electron microscopy work by Hu and Rijkenberg [9] identified important time points in infection structure formation by *P. triticinia* on susceptible and resistant lines of hexaploid wheat. Six hours after infection, the fungus forms appressoria over stomata openings. After12 hours, the fungus has successfully penetrated into the stoma, formed substomatal vesicles (SSV), and primary infection hyphae are visible. After SSV formation, the primary infection hypha grows and attaches to a mesophyll or epidermal cell. At 24 hours postinoculation (HPI), a septum appears separating the haustorial mother cell from the infection hypha after which the fungus forms haustorium and penetrates the cell [9].

Much remains unknown regarding the molecular basis of disease development. Nevertheless, numerous genes involved in the wheat-pathogen response have been identified. These include genes encoding leaf rust resistance [2], antifungal hydrolases such as glucanase and chitinase [10, 11], protein kinases [12], and enzymes involved in the production of reactive oxygen species [10, 13]. Of the leaf rust resistance genes, *Lr1* is described as one of those conferring resistance to leaf rust low pathogenic races in wheat seedlings [2, 14].

Until recently, however, molecular studies of the host response to pathogen attack were restricted to the analysis of a relatively small number of genes or proteins. This has changed with the development of high-throughput technologies, such as cDNA and oligonucleotide microarrays that allow expression profiling of thousands of genes simultaneously. Thus, microarrays represent important tools for the global analysis of many plant processes, including the response to pathogen attack [15–20]. There are already a number of studies that have used cDNA and oligonucleotide microarrays to characterize plant-pathogen interactions. *Arabidopsis* cDNA microarray analysis demonstrated a substantial network of regulatory coordination among different defence signaling pathways [21]. In another study, microarray analysis of the *Arabidopsis* transcriptome during systemic acquired resistance revealed groups of genes with common regulation patterns [22]. Microarrays have also been used to investigate plant defence in nonmodel organisms. For instance, oligonucleotide arrays were used to analyze maize challenged with the pathogen *Cochliobolus carbonum* [23], barley challenged by *Blumeria graminis* f. sp. *hordei* [18], and *Fusarium* [24] to identify genes that showed expression changes during the interactions. In wheat lines carrying the leaf rust resistance gene *Lr1,* however, little is known regarding the expression of genes during differential responses following inoculation with compatible and incompatible races of the pathogenic leaf rust fungus *P. triticina*. In this report, we describe the development and use of a wheat cDNA microarray to examine changes in gene expression in the wheat line RL6003 in response to challenge by compatible and incompatible races of *P. triticina*.

## 2. MATERIALS AND METHODS

### 2.1. Plant material and inoculations

Wheat near isogenic line RL6003 (Thatcher *6/Centenario), carrying the *Lr1* leaf rust resistance gene, was used throughout the study because Thatcher is known to strongly express the introgressed *Lr* genes. Seeds were sown in 12 cm diameter plastic pots filled with a 3 : 1 soil/Sunshine Professional Growing Mix 5 soil mixture (Sun Gro Horticulture, Vancouver, British Columbia, Canada), at a rate of one seed per pot. Seedlings were maintained in a growth room at 20°C with a 16-hour photoperiod and a photon flux density of approximately 145 *μ*mol m^−2^s^−2^. They were watered and fertilized as required. Plants were inoculated at the 2-3 leaf stage with urediniospores of *P. triticina* virulence phenotypes BBB (incompatible) or TJB (7-2) (compatible) [25] mixed with a light mineral oil (Bayol-Esso Canada, Oak Bluff, Manitoba, Canada). Twenty five *μ*l of a 16.6 mg uredinispores/ml of mineral oil inoculum were applied to each pot. Seedlings were sprayed until runoff with the spore suspensions using a deVilbis-type sprayer, connected to an air line and operated at a pressure of 17 KPa. Control plants were sprayed only with mineral oil, referred to as mock inoculation. The inoculated plants were allowed to dry for at least 30 minutes to let the oil volatilize and then incubated overnight in a 100% humid chamber (Percival model I-60D) (Percival Scientific, Perry, Iowa, USA). They were subsequently transferred to a growth chamber and kept under the conditions described above. Leaf tissues were sampled 3, 6, 12, and 24 HPI. Plants were rated for symptom development 12 days after inoculation [25]. Three independent biological replicates were performed.

### 2.2. Microarray preparation

Four cDNA libraries were constructed from either Thatcher *Lr1* leaf tissue (4-leaf stage) sampled 24 hours after inoculation with *P. triticina* race BBB or developing seeds of “Glenlea” sampled at 5, 15, or 25 days postanthesis. Plasmid DNA, pBK-CMV (Stratagene, La Jolla, Calif, USA), and pSport 6.0 (Invitrogen, Burlington, Ontario, Canada), respectively, for the leaf and seed libraries, were isolated using the Perfect Prep Direct Bind Kit (Eppendorf, Hamburg, Germany), adapted for the Qiagen 3000 Liquid Handling Robot (Qiagen, Mississauga, Ontario, Canada). EST sequencing was performed with M13F and M13R universal primers using the Big Dye V2.0 (ABI, Foster City, Calif, USA) and resolved on an ABI 377 Genetic Analyzer. High-quality sequence was obtained using the softwares phred and cross_match (default settings except for minscore = 20 and minmatch = 12) [26]. A total of 24 959 ESTs were obtained from the libraries and were submitted to GenBank (accessions BE417910-BE418911, BG903972-BG910140, BQ619578-BQ620868, BQ235898-BQ252370, ES316459). The sequences were assembled using CAP3 software (default parameters except overlap length cutoff = 40 and overlap identity cutoff = 95) [27], and in combination with some manual annotations, a unigene set of approximately 12 500 sequences was identified. Plasmids of the unigene set members were used as template for PCR amplification of the inserts using universal M13 primers. Amplicons were visualized on agarose gels and selected for use on the microarray based on their quality and uniqueness. A subset of 7728 selected amplicons was consolidated to 384-well plates and purified using Multiscreen 384 PCR filtration plates (Millipore, Nepean, Ontario, Canada). Aliquots of the purified products were run on 1% (w/v) agarose gels, sized and quantified using known amounts of lambda *Hind* III markers. They were then diluted in 1 × microspotting plus solution (TeleChem, Sunnyvale, Calif, USA) to a final concentration of 100 ng/*μ*L. A total of 384 controls provided with the SpotReport-3 (Stratagene) kit, consisting of *Arabidopsis* CAB, rbcL and RCA genes, human *β*-cell actin gene, cot-1, ssDNA and polyA, as well as wheat CAB, rbcL, RCA, and human *β*-cell receptor genes, were also included as were 96 empty wells. All 8208 samples were printed by Telechem onto SuperAmine substrates according to the company standard procedures (www.arrayit.com) in 48 blocks of a 19 × 18 array using side-by-side double spotting.

### 2.3. RNA extraction and preparation of fluorescent probes

Approximately 1 g of leaf tissue was frozen in liquid N_2_ and ground to a fine powder with a mortar and pestle. Total RNA was extracted using the RNAWiz isolation reagent (Ambion, Austin, Tex, USA) and fursther purified using RNeasy columns (Qiagen), according to manufacturer's instructions.

Fluorescent probes were synthesized by reverse transcription of the RNA in the presence of Cy3-dCTP or Cy5-dCTP (Amersham Biosciences, Baie-d'Urfé, Quebec, Canada), using a modification of the protocol for the preparation of unamplified cDNA [28]. Each labeling reaction was performed in a 40 *μ*L volume containing 10 *μ*g total RNA, 0.75 *μ*g oligo(dT)12–18 (Invitrogen), 500 *μ*M each of dATP, dGTP, and dTTP, 50 *μ*M dCTP, 25 *μ*M of either Cy3-dCTP or Cy5-dCTP, 8 *μ*L of 5 × SuperScript II first strand buffer (Invitrogen), 10 mM dithiothreitol, and 40 U of RNasin RNase inhibitor (Promega, Madison, Wis, USA). The reaction mixture was heated at 65°C for 5 minutes and then cooled to 42°C. Four hundred units of SuperScript II Reverse Transcriptase (Invitrogen) were added, and the labeling reaction was allowed to proceed for 2 hours at 42°C. The RNA was then degraded by treatment with 5 *μ*L of 50 mM EDTA (pH 8) and 2 *μ*L of 10 N NaOH at 65°C for 20 minutes. After the addition of 4 *μ*L of 5 M acetic acid, the mock and TJB (MT) or mock and BBB (MB) samples were pooled, and the cDNA was precipitated by the addition of one volume of isopropanol. The pellet was washed with 70% ethanol, air dried, resuspended in 5 *μ*L of nuclease-free water, and added to 60 *μ*L of DigEasy Hyb solution (Roche, Indianapolis, Ind, USA) containing 0.45 *μ*g of yeast tRNA and 0.45 *μ*g of denatured salmon sperm DNA. Targets were heated (65°C) prior to adding to the microarrays.

### 2.4. Hybridization

Slides were prehybridized by incubating for 45 minutes at 42°C in a preheated solution of 5 × SSC, 0.1% (w/v) sodium dodecyl sulfate, and 1% (w/v) bovine serum albumin. They were then washed in water and isopropanol and allowed to air dry. Denatured dual Cy3/Cy5-labeled target cDNAs were added directly to each slide and sealed under a 22 mm × 60 mm hydrophobic coverslip (Sigma-Aldrich, Oakville, Ontario, Canada). Microarray slides were placed on top of support slides in a slide staining box (Diamed, West Chester, Pa, USA) containing approximately 20 mL DigEasy Hyb at the bottom, and incubated at 42°C for 18 to 20 hours. After hybridization, the slides were quickly rinsed with 1 × SSC (until coverslips fell off), washed three times with 1 × SSC and 0.1% SDS (w/v) for 10 minutes at 50°C, then washed three more times with 0.1 SSC for 1 minute at room temperature. The slides were dried by centrifugation and scanned for fluorescence emission using a GenePix 4000B scanner (Axon Instruments, Union City, Calif, USA). Photomultiplier (PMT) voltages were adjusted manually to balance the amount of red and green signals in the images obtained.

### 2.5. Microarray data analysis

Feature intensities were quantified using GenePix Pro 5.1 array analysis software and Acuity 4.0 (Axon Instruments). Microarray spots flagged as “bad,” “not found,” or with specific unwanted feature parameters were removed automatically by imposing stringent filtering criteria to the entire microarray dataset (acuity criteria dia ≥60 and circularity ≥80). Lowess normalization was applied to the data. The log_2_ median (logM) ratio of expression values for genes, incompatible versus mock (MB) and compatible versus mock (MT), that met the filtration criteria and were found in both treatment sets, was subjected to further statistical analysis. An analysis of variance using the SAS procedure Mixed (SAS, Cary, NC, USA) was carried out on the (logM )ratio data for incompatible (MB) and compatible (MT) interactions, and contrasts were used to identify genes with significant differences in expression at one or more time points as defined by their *P* values. The model included compatibility type (MB or MT), time, and the interaction between compatibility type and time. Main plots were replicate by interaction type (representing MB or MT pairs of batches of inoculum) in a randomized complete block arrangement, and subplots were individual slides. Genes with a significant *F* value (*F* ≤ .055) for a contrast between interaction type by time were selected and verified by resequencing the DNA used to create the spot on the microarray. In addition to the ANOVA, a *t* test was used to determine at which time point(s) the treatments yielded significantly different expression values (*P* ≤ .05).

### 2.6. Cluster analysis and annotation

Putative gene function was assigned based on BLASTX analysis against the GenBank nonredundant protein database [29]. The best hit with an *E* value less than or equal to 1 × 10^−5^ was used to assign the EST annotation. In some cases, the second best hit had a more informative description of the gene function and was used to assign an annotation. If a significant BLASTX hit could not be found, then the annotation from the Dana-Farber Cancer Institute (DFCI) Wheat Gene Index cluster that contained the EST was used http://compbio.dfci.harvard.edu/tgi/, if available. Gene ontology (GO) annotation was performed by using BLASTX to compare wheat genes to the predicted protein sequences for *Arabidopsis thaliana* from The *Arabidopsis* Information Resource (TAIR) Genome Release Version 6. The best hit with an *E* value less than or equal to 1 ×10^−5^ was used to assign the ESTs an *Arabidopsis* homolog and the TAIR annotation tool was used to place the genes into GO categories.

## 3. RESULTS

### 3.1. Pathosystem selection and disease expression

**Figure 1:**
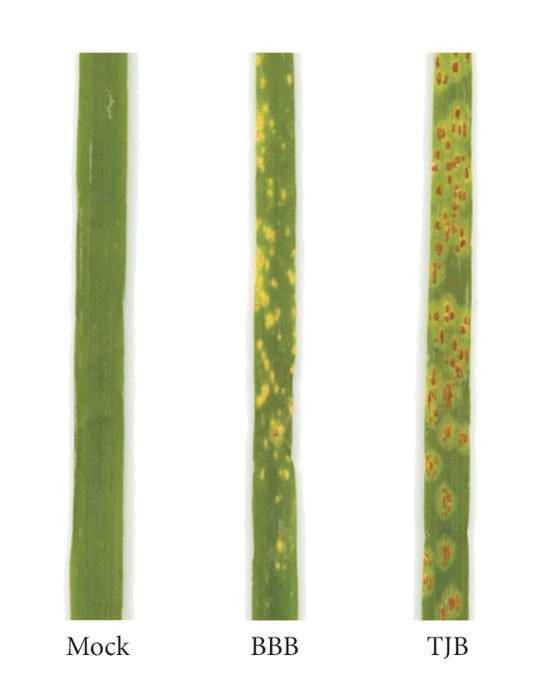
Phenotypic reaction of wheat near-isogenic line RL6003 to mock-inoculation with oil or inoculation with avirulent race 1 (BBB) or virulent race 7-2 (TJB) of *Pucinia triticina*. An incompatible interaction showing a “;1-” infection type with very small pustules and no sporulation (BBB), and a compatible interaction showing a “3+ 4-” infection type with large pustules and abundant sporulation (TJB) are illustrated.

To unravel global changes in the host transcriptome during leaf rust interactions, wheat RL6003, which carries the *Lr1* resistance gene, was challenged with two different races of the leaf rust fungus, *Puccinia triticina*. Virulence phenotype BBB carries the avirulence gene *Avr Lr1* and provokes an incompatible reaction on RL6003, while race TJB lacks the avirulence gene and therefore produces a compatible interaction. A sharply contrasting phenotype was observed between the two interactions. The TJB interaction displayed a compatible “3+ 4-” infection type characterized by large pustules and abundant sporulation while interaction with BBB yielded an incompatible “;1-” infection type (Figure 1) [25]. The presence of small pustules or no sporulation was typical of a hypersensitive response (HR). No symptoms were observed on mock-inoculated seedlings (Figure 1).

### 3.2. Gene expression profiling

To identify genes whose expression profile can distinguish compatible from incompatible interactions, we designed an experiment using the pathosystem outlined in the previous section: one host genotype, RL6003, inoculated with the two rust races, BBB and TJB, each compared to a mock control sample. Infection ensued and samples were taken at four time points: 3, 6, 12, and 24 HPI. The whole experiment was repeated three times to generate three biological replicates. Sequences on the microarray were taken from an assembly of approximately 12 500 unigenes generated from an EST set of 24 959 sequences. Parameters to the assembly program, CAP3, were the same as those used by TIGR for its unigene assemblies (www.tigr.org). These parameters favoured assembly of homoeologous genes into single sequences without the inclusion of paralogous genes. A total of 5728 microarray “spots” met the filtration criteria outlined in the materials and methods, 4439 of which were shared between the MB and MT datasets and were used for statistical analysis. Statistical analysis of the microarray data using SAS and confirmation of the identity of ESTs by sequencing revealed 192 genes that were significantly differentially regulated across at least one of the four times points between the compatible and incompatible interactions (*P* ≤.055 for the pathogen type by time interaction ANOVA effect)Supplementary Table 1 available online at doi:10.1155/2007/17542). Seventy one percent of the genes had different expression values between the two treatments, as determined using tests (*P* ≤ .05), at a single time point, 18% of genes showed differential expression at 2 time points, and 1% at 3 time points, while no genes in this set were differentially expressed at all four of the time points investigated. At 6 HPI, 46% of the genes were differentially expressed, followed by 40% at 24 HPI. The 3- and 12-hour time points showed much less differential gene expression at 11% and 10% of the genes, respectively.

### 3.3. Gene annotation

**Figure 2:**
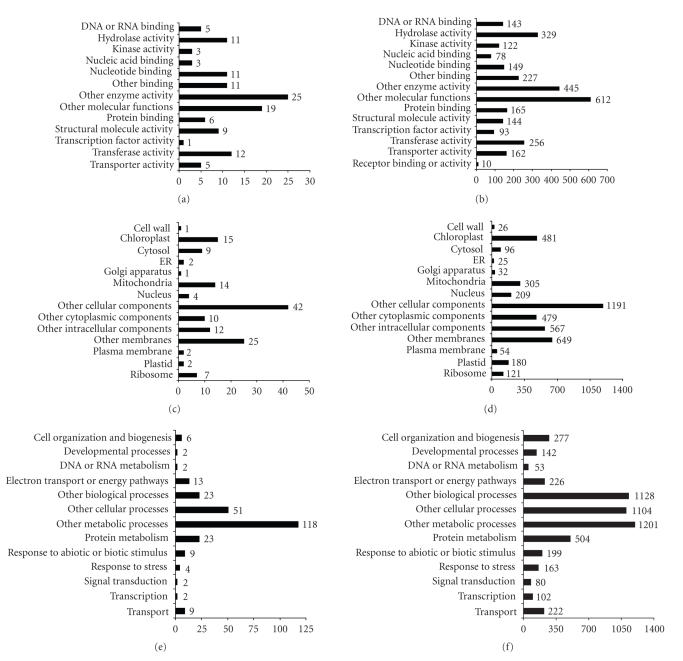
Gene ontology assignment of differentially expressed genes. A BLASTX search of the differentially expressed sequences and all of the sequences on the microarray against the set of predicted *Arabidopsis thaliana* proteins was used to assign gene ontology. The first hit with an *E* value less than or equal to 1 × 10^−5^ was used as a functional assignment and the TAIR GO annotation tool was used to bin the genes into the ontology groupings: (a) molecular function of the differentially expressed genes; (b) molecular function of the genes on the microarray; (c) cellular location of differentially expressed genes; (d) cellular location of genes on the microarray; (e) biological process for differentially expressed genes on the microarray; (f) biological process for the genes on the microarray.

Using BLAST analysis, we were able to assign, with various degrees of confidence, a potential function to 150 of the differentially regulated genes. Forty two gene sequences did not share sequence homology to any sequences in the GenBank nonredundant protein database at our threshold *E* value (1 × 10 − 5), and therefore, no annotation could be assigned. Gene ontology (GO) annotation was performed using the TAIR annotation tool and the genes were placed into GOSlim categories (Figure 2). Molecular function assignment revealed members in a broad range of categories (Figure 2(a)) including those genes with putative hydrolase activity, nucleotide binding activity, and protein binding activity. The differentially regulated genes were also members of a variety of cellular components with a large representation from the chloroplast and mitochondria (Figure 2(b)). The biological process GO assignment illustrated that genes involved in protein metabolism, electron transport or energy pathways, and response to abiotic/biotic stimuli were well represented in our dataset (Figure 2(c)).

Of the 7728 cDNAs spotted on the chip, 2142 came from the *P. triticina* challenged EST library raising the possibility that some of the cDNAs spotted to the array were fungal in origin. Of the 2142 ESTs, only 2 sequences found a significant match (*E* value < 1 × 10^−5^) against the phytopathogenic fungi and oomycete EST database available from COGEME (http://cogeme.ex.ac.uk) and had no match against ESTs from non-*P. triticinia* challenged plant EST libraries (est_others from GenBank; http://www.ncbi.nlm.nih.gov). Neither of these 2 sequences was found to be differentially expressed in this study. Comparison of the 2142 ESTs to an in-house *P. triticinia* library of over 40 000 ESTs (Bakkeren, unpublished) found that 22 had significant matches to fungal sequence using BLAST (*E* value < 1 × 10^−5^). Of these, only one was found to be differentially expressed in this study, and closer examination revealed that the sequence (TaLr1013E06R) was a gene from a conserved pathway and had stronger homology to a rice gene than to the fungal sequence.

### 3.4. Genes involved in photosynthesis

**Figure 3:**
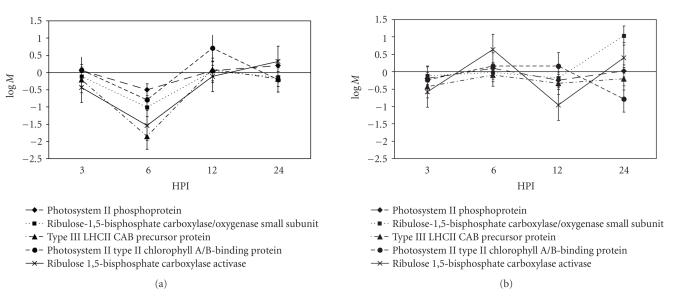
Expression profile of several genes involved in photosynthesis. Panel (a) incompatible, panel (b) compatible interaction. Genes involved in photosynthesis share a similar expression profile between the two treatments. At 6 HPI, the incompatible interaction shows a general reduction in the expression of some photosynthetic genes. The *y*-axis is the logM value, the log_2_ median ratio of expression values for genes from incompatible versus mock inoculations (MB) or for genes from compatible versus mock inoculations (MT). Error bars are the standard error for the expression of that gene in our mixed linear model as determined by the SAS procedure Mixed.

A number of genes involved in the energy status of the cell were differentially regulated. For example, the interaction between RL6003 and an incompatible or compatible rust race produced a differential expression pattern for ribulose 1,5-bisphosphate carboxylase/oxygenase (Rubisco) small subunit (TaLr1140F12A, TaLr1150F09F). Rubisco is the main source of energy production for the plant cell, generating ATP and reductive potential (NADPH) through photosynthesis. In the incompatible interaction, the expression of the gene was reduced at 6 HPI but returned to levels identical to the mock inoculation at 12 and 24 HPI. The compatible interaction, however, was characterized by a strong increase in the transcription of the Rubisco small subunit at 24 HPI (Figure 3).

Along with differential expression of Rubisco, there was a change in the transcription of its major regulatory protein, Rubisco activase (TaLr1173G10F, TaLr1175G08F) [30]. At 3 HPI, there was a small reduction in the transcription of Rubisco activase relative to basal levels for both the compatible and incompatible interactions. At 6 HPI the plants involved in an incompatible interaction continued to repress the expression of Rubisco activase, while in the compatible interaction, expression of the transcript returned to near basal levels. Other genes in the photosynthetic apparatus were differentially expressed as well. Chlorophyll A/B-binding protein genes (TaLr1131B12A, TaLr1142E01A, and TaLr1167E07A), an FtsH like AAA-metalloprotease (TaLr1134F10F), and a photosystem II phosphoprotein (TaLr1130A12A) were down regulated at 6 HPI in the incompatible interaction relative to basal and compatible interaction levels. In both treatments, the expression of these genes was at basal levels for the other time points. An important enzyme involved in photorespiration, glycolate oxidase (TaLr1162D03A), was also found to be differentially regulated. Its expression was repressed at 3 and 6 HPI in plants involved in an incompatible interaction but not in a compatible one.

### 3.5. Genes involved in redox control

**Figure 4:**
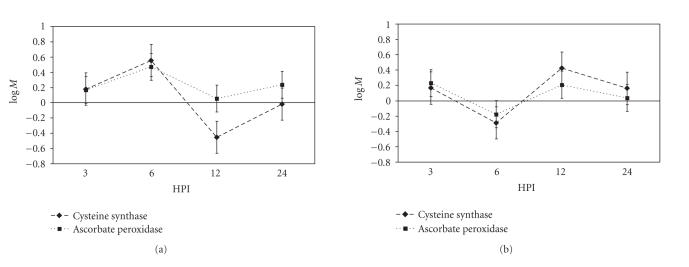
Transcription of genes involved in ROS scavenging. Two genes involved in reducing the concentration of reactive oxygen species in the cell show differential expression between the two treatments: (a) incompatible, (b) compatible. Error bars are the standard error for the expression of that gene in our mixed linear model as determined by the SAS procedure Mixed.

The production of reactive oxygen species (ROS) is central to the defence mounted by plants in reaction to challenge with an incompatible pathogen [31, 32]. Shortly after challenge, the plant produces an “oxidative burst” which is believed to have three roles: (i) induces damage on the invading organism, (ii) bolsters structural defences, and (iii) acts as a biochemical signal to induce other defence mechanisms in the plant. The production of ROS presents a challenge to a plant because of the damage these compounds can cause to its own proteins, DNA, and other cellular components. The plant must therefore invoke a balanced system that produces ROS for defence at the same time as a number of antioxidants to protect against oxidative damage. Ascorbate peroxidase (APX: TaE25014D09F), a central component of the ROS scavenging system, was differentially expressed in the compatible and incompatible interactions. At 6 HPI, transcription of APX in plants challenged by the incompatible race was significantly upregulated relative to transcription in those challenged by the compatible race (Figure 4).

Another critical protein for maintaining redox balance in the face of oxidative stress is glutathione, which acts as ubiquitous supplier of reduction power for cellular processes. Glutathione is a conjugate of cysteine and glutamate and therefore relies on both of these amino acids for synthesis. Cysteine synthase (TaE25012D06R) is the final catabolic step in the production of cysteine and was found to be differentially regulated between the two treatments (Figure 4). Plants in an incompatible interaction increased the transcription of this gene at 6 HPI, while those in a compatible interaction reduced it. At the 12-hour time point, the incompatible plants had reduced transcription of the gene relative to basal levels while the compatible plants had upregulated transcription.

### 3.6. R-and related genes

Recognition of *P. triticina* infection by RL6003 occurs through a gene-for-gene system where resistance only occurs if the plant possesses an R-gene encoding a product able to detect the presence or action of a specific avirulence factor (Avr-gene) produced by the pathogen [33–35]. If either of these components is missing, the plant is unable to mount an effective defence against the invader. R-genes identified to date fall into five different categories and commonly contain a leucine-rich repeat region (LRR), a domain known to be involved in protein-protein interactions [33]. A significant portion of the microarray cDNA content used in this study was derived from a library of RL6003 (Thatcher *Lr1* ) leaf tissue collected 24 HPI with an incompatible *P. triticina* race. Although we have recently confirmed (by cloning of *Lr1* in a separate study) that *Lr1* was not present on the array (unpublished data), three of the differentially regulated genes identified in our experiment nevertheless contained LRR domains, two of which are of the NBS-LRR class of R-genes (TaE05012C12F, TaLr1134C01R). There did not appear to be a coordinated pattern of expression among these three genes at the time points tested (Supplementary Table 1).

**Figure 5:**
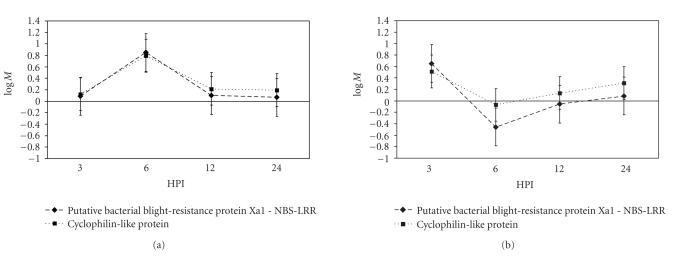
An *Xa1*-like NBS-LRR and a cyclophilin gene have similar transcription patterns. At 6 HPI, both of these genes were differentially expressed in the two treatments: (a) incompatible, (b) compatible. The coexpression of these genes may indicate that a pathogen elicitor recognition system similar to the one recently identified in *Arabidopsis* exists in wheat. Error bars are the standard error for the expression of that gene in our mixed linear model as determined by the SAS procedure Mixed.

Recently, a cyclophilin was found to be necessary for host-pathogen recognition in *A. thaliana* [36]. A cyclophilin-like protein (TaE25043B07R) was differentially regulated in our experiment and it shared an expression profile very much like that of one of the identified NBS-LRR genes (TaE05012C12F, Figure 5).

**Figure 6:**
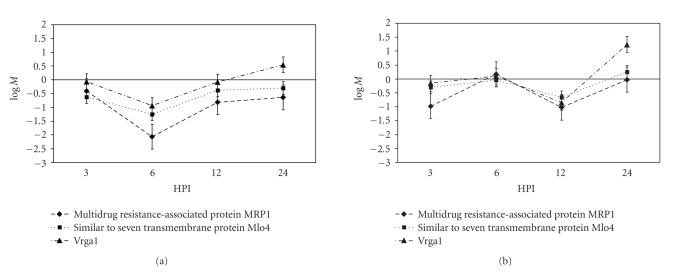
Coordinated expression of disease response genes. Genes involved in the disease response in other plant hosts are differentially expressed between the two treatments: (a) incompatible, (b) compatible interaction. Transcription of each of the three genes was reduced at 6 HPI in plants challenged with the incompatible race. The *Mlo4*- and *Vrga1*-like genes were differentially regulated at 24 HPI, with transcription increasing in the plants challenged with the incompatible race. Error bars are the standard error for the expression of that gene in our mixed linear model as determined by the SAS procedure Mixed.

Two other disease-associated genes were also identified, an *Mlo4*-like gene (TaLr1140F02A) and a multidrug resistant-associated protein 1-(*MRP1-*) like gene (TaLr1143B08F). These two genes showed similar expression profiles in the incompatible interaction. They were generally repressed in this treatment, with the strongest repression at 6 HPI. In the compatible interaction, initial repression of these genes occurred at 3 and 12 HPI, but returned to near-basal levels at the other time points (Figure 6).

### 3.7. Other pathways affected by disease progression

Modification of proteins with ubiquitin molecules serves many purposes in cellular metabolism [37]. Our study identified eight genes that play a role in the ubiquitination cascade. These include ubiquitin associated proteins (TaE05010C02F, TaE15029B06R, and TaE012C02A), a ubiquitin conjugating enzyme (TaE25042D03A), and components of ubiquitin ligase complexes (TaE05014D09F, TaE15001B10R). There did not appear to be a coordinated expression pattern among the differentially expressed ubiquitination components.

In addition to genes already mentioned, a large collection of genes with an identified role in disease or stress response were significantly differentially regulated between plants challenged with incompatible and compatible races of *P. triticina*. Components of the shikimate-phenylpropanoid pathway, caffeoyl-CoA-O-methyltransferase (TaE05029C04F), 3-deoxy-D-arabino-heptulosonate 7-phosphate synthase (TaLr1013E06R), farnesyl-pyrophosphate synthetase (TaLr1161D04R), and UDP-glucose glucosyltransferase 1 (TaLr1148B05F), revealed differential transcription patterns as did genes involved in signal transduction (myb-like transcription factor TaE05012H07F, WRKY11 transcription factor TaLr1159C08F, calmodulin TaLr1021E02R, mitogen activated protein kinase kinase TaLr1106B01R, and phosphatidylinositol-4-phosphate 5-kinase-like gene TaE05025C08F), and stress-associated genes such as heat shock proteins (HPS70—TaE05013A12R, HSP80—TaE05005C08A), osmotic control genes (betaine aldehyde dehydrogenase—TaE25007B01R, S-adenosylhomocysteine hydrolase 2—TaLr1174G05R, CLC-f chloride channel—TaE25020B11R), and genes of unknown function induced by stress in other organisms.

## 4. DISCUSSIONS

To gain insight into the transcriptional changes that occur in incompatible and compatible reactions of the wheat isogenic line, RL6003, challenged with avirulent and virulent races of the leaf rust pathogen *P. triticina*, we conducted a cDNA microarray gene-profiling experiment. In this study, we used an ANOVA to identify 192 wheat genes that showed differential expression profiles resulting from two different pathogen challenges. Our results revealed a clear differentiation between incompatible and compatible interactions and led to the identification of differentially regulated genes putatively involved in defence reactions.

We examined four time points early in the infection stages of virulent and avirulent races of *P. triticinia* on the wheat line RL6003. At 6 and 24 HPI, 46% and 40% of the differentially expressed genes, respectively, were found to have different degrees of change between the two pathogen treatments. In contrast, only 11% and 10% of genes were differentially expressed at 3 and 12 HPI. Upon inoculation, *P. triticinia* urediospores germinate within hours on the wheat leaf surface, form a germ tube, and grow until a stomatal guard cell is reached. At this point, the fungus forms an appressorium over the stoma, enters by force into the substomatal space, and creates a substomatal vesicle [38]. The fungal growth into the intercellular space occurs at 6 HPI [9] and coincides with the highest differential gene expression in our study. At 24 hours, when 40% of the identified genes reveal changes in expression, the fungus has already produced a septum which separates the primary infection hypha from the newly formed haustorial mother cell which initiates contact with adjacent mesophyll or epidermal cells as shown by Hu and Rijkenberg [9]. Those authors have studied the infection process of *P. triticinia* on susceptible (Thatcher) and resistant wheat lines (RL6040 which contains the *Lr19* leaf rust resistance gene and RL6043 which contains the *Lr21* leaf rust resistance gene) of wheat and no significant differences were observed between the three lines during these early infection events. Moreover, very similar results were obtained during *P. triticinia* infection of nonhost species such as maize, oat, sorghum, and barley [39]. Based on these studies, it is likely that the growth of the two races of *P. triticinia* used on RL6003 in this study invoke very similar responses during the early infection stages as that of the *P. triticinia* races used by Hu and Rijkenberg on other resistant wheat lines [9]. If this is the case, RL6003 was responding to the leaf rust pathogen before any cellular penetration had occurred. This suggests that the plant is able to perceive fungal movements on the leaf surface and past the guard cells, possibly through cell membrane rearrangements or cell wall degradation products caused by compounds excreted by the fungus. Our early stage data for differential expression of host genes contrast the findings of Caldo et al. [18] where no differences in expression among barley genes were observed up to 16 HPI after treatment with *Blumeria graminis* f. sp.*hordei*. Those authors found differential gene expression only during the stages involving haustoria-plant epidermal membrane contact. However, differential plant gene expression has been detected in other plant systems during the initial hours of pathogen infection [19, 40, 41]. This study is therefore the first to show such early differential gene expressions in wheat using the pathogen *P. triticinia*.

Resistance of wheat genotypes to leaf rust pathogens can often be attributed to a gene-for-gene interaction [25]. In general, its visible defence response, the hypersensitive response (HR), reflects a multitude of metabolic changes in affected cells [42, 43] and results in localized plant cell death. RL6003 challenged with an incompatible race of *P. triticinia* exhibits an HR (Figure 1) and it is believed to be mediated through pathogen elicitor detection by *Lr1*. One of the hallmarks of the HR disease response is the generation of reactive oxygen species (ROS) in an oxidative burst across the plasmalemma of plants cells, perturbing the redox state of cells and allowing controlled oxidation, which may have an immediate antimicrobial effect [43–45]. In *Asparagus sprengeri* mesophyll, HR induction by a G-protein activator, mastoparan (MP), resulted in significant changes in photosynthesis [46]. The authors demonstrated that during the elicitor-induced HR and oxidative burst, light was stimulating the HR, that O_2_ evolution ceased due to a disruption of photosystem II (PSII) and electron transport and that photosynthesis was eventually inhibited by MP [46]. Recent work to identify ESTs involved in a rice-rice blast fungus interaction found that transcription of photosynthetic genes, such as ribulose 1,5-bisphosphatase carboxylase (Rubisco), photosystem I-(PSI-) associated genes, PSII-associated genes, and chlorophyll A/B-binding protein (CAB) genes, were suppressed in both resistant and susceptible interactions [40]. In soybean, it has been shown that in addition to known defence regulated genes, challenge with *Pseudomonas syringae* induced a rapid downregulation of photosynthesis and that this effect was HR specific [41]. In their study, Zou et al. [41] found that chloroplast-related genes had reduced expression 8 hours after challenge and found a decrease in PSII activity and an interruption in the photosynthetic electron transport chain. It is speculated that the over-reduction of PSII components and an interruption of electron transport result in electron leakage and the formation of ROS [19, 41, 46]. Our data also identified photosynthesis-related genes that were downregulated during pathogen challenge (Figure 3). Six hours after inoculation, we observed a coordinated decrease in transcription of these genes in the resistant but not susceptible interactions. If this decrease in photosynthetic gene expression is linked or due to the HR as observed in other studies [40, 41, 46, 47], one may reasonably ask “what would be the purpose”? Six hours into the infection, the fungus has just entered the substomatal space and has yet to directly invade a mesophyll or epidermal cell. If the decrease in photosynthesis is used to generate ROS for an oxidative burst, then this burst does not likely have any antimicrobial activity since *P. triticinia* growth is identical in the first 24 hours of infection in susceptible and resistance reactions with wheat [9]. One of the key enzymes involved in removing H_2_O_2_, APX [45], was identified as being differentially expressed in our study. Although APX has been shown to be translationally inhibited during programmed cell death [48], based on its expression profile in this study, we speculate that the cellular H_2_O_2_ concentration increased 6 hours after challenge with an incompatible race of *P. triticinia* and that APX levels were likely increased in an attempt by the cell to curtail oxidative damage. If ROS are generated at this stage, it is more likely that they act on nonhost defence responses and have a role in cellular fortification, intercellular signaling, or act to change the redox state of the cell for regulatory purposes. Additional experiments to measure ROS generation during the wheat-rust interaction would be needed to determine if an oxidative burst is occurring at 6 HPI.

The interruption in the electron transport chain in the chloroplast during the HR is believed to be mediated by a Zn-dependent AAA-metalloprotease called FtsH. During stress, a component of PSII, D1, can become damaged and has to be replaced by a newly synthesized D1 protein in order to restore PSII function. FtsH degrades damaged D1, allowing for a functional version of the protein to take its place [49, 50]. We identified a wheat AAA-metalloprotease FtsH-like gene (TaLr113410F) with reduced expression in the incompatible reactions at 3 and 6 HPI. This may indicate that PSII is affected during *P. triticinia* pathogenesis. Similar results were reported by Seo et al. [47] in tobacco where the expression of a chloroplast FtsH protein was reduced and electron transport interrupted 6 hours following the induction of an HR by tobacco mosaic virus. Those authors believed that the disruption of photosynthesis perturbed cellular homeostasis (possibly by consuming reductive power) which accelerated the HR. They suggested that it was also possible that reducing photosynthesis reduced sugar production, limiting the food source for the invading pathogen. It seems likely that the downregulation of photosynthesis and the interruption of the electron transport chain may be conserved features of the defence response to pathogen challenges as it is shared by rice [40], tobacco [47], and soybean [41] and we observed evidence of this mechanism in wheat.

Plant responses to biotic stress involve multiple interlinked regulatory pathways that transduce suitable signals for efficient defence reactions [33, 34, 42, 51, 52]. In addition to the ROS pathway described earlier, our microarray profiling detected a number of genes in the shikimate and phenylpropanoid pathways that were differentially regulated. The shikimate pathway leads to the biosynthesis of phenylpropanoid, phytoalexins (terpenoids, flavonoids), lignin, and salicylate which are main secondary metabolites of the disease resistance machinery [18, 53– 56]. Our analysis identified that 3-deoxy-D-arabino heptulosonate-7-phosphate synthase, the entry point into the shikimate pathway, was differentially regulated between the two pathogen treatments. At 6 HPI, transcription was found to be increased in the incompatible interaction but the greatest difference occurred at 24 HPI when the expression was increased in the compatible interaction and downregulated in the incompatible interaction. Other components downstream in the shikimate-phenylpropanoid pathway were also differentially regulated, such as 3-beta hydroxysteroid dehydrogenase-isomerase, which is involved in metabolism of alkaloids, caffeoyl-CoA O-methyltransferase, which is involved in lignin biosynthesis and cell wall fortification, and farnesyl-pyrophosphate synthetase, which is involved in the biosynthesis of terpenoid compounds. All of the genes identified in the shikimate-phenylpropanoid pathway had their strongest expression in plants challenged with a compatible pathogen. This may illustrate an important distinction in the *Lr1* pathosystem in which the plants involved in an incompatible interaction dedicate cellular resources to redox systems, as evidenced by early expression of ROS enzymes and downregulating photosynthetic genes, while plants in a compatible interaction trigger nonspecific defence responses, such as cellular fortification and alterations in their secondary metabolism.

In our data, we identified three genes that contain LRR domains, regions involved in protein-protein interactions, and two of the three were of the NBS-LRR class, with homology to putative R-genes, although neither of them encodes the *Lr1* gene (unpublished data). One of the NBS-LRR genes (TaE05012C12F) shares homology with the rice protein *Xa1*, which confers bacterial blight resistance [57]. Interestingly, Yoshimura et al. [57] showed that *Xa1* expression was induced by wounding and pathogen attack, which prompted the authors to speculate that *Xa1* may be involved in enhancing the disease response to bacterial blight. Although it is not responsible for AVR *Lr1* recognition, this *Xa1*-like NBS-LRR gene may be activated to serve a similar purpose, which is the enhancement of the defence response; it is possible that other, yet undescribed, fungal factors present in race BBB interact with this resistance-like gene. Plants in the incompatible interaction showed a spike of induction of this gene at 6 HPI, which coincided with the expression of other defence systems genes. It was also recently found that a cyclophilin protein plays a role in an R-gene signaling pathway [36]. The bacterial effector, AVRRPT2 from *Pseudomonas syringae, * relies on an *Arabidopsis* cyclophilin to initiate its protease activity. Once activated, AVRRPT2 cleaves RIN4, which is then recognized by the NBS-LRR protein RPS2 and triggers an incompatible response [36]. In our study, a wheat cyclophilin had a nearly identical expression pattern to that of the *Xa1*-like gene (Figure 5) and may indicate that they operate in the same pathway and that this method of avr-gene activation described in *A. thaliana* exists in wheat.

The recognition of invading pathogens activates a signal cascade that leads to a change in gene expression. A number of important components in the transduction of stress and disease signals have been identified. These include several mitogen-activated protein (MAP) kinases, MAP kinase kinases, and transcription factors [58, 59]. Our data revealed coordinated expression among components involved in MAP pathway signaling, transcription factors, and genes that have been found to be expressed during a disease response. Their coordinated expression may indicate that these genes operate in the same signal transduction pathway.

Plant defence response to pathogen attack can also include the modification of proteins by ubiquitin [60, 61]. Many members of the ubiquitination cascade were found differentially regulated in our experiment, including ubiquitin activating enzymes, ubiquitin conjugating enzymes, and components of SCF ubiquitin ligase complexes. Despite the lack of a unified expression profile among the members of the pathway, it is clear that the process of protein modification by ubiquitin plays a role in the disease response of wheat to races of *P. triticinia*.

There were a number of genes identified in this study that have protein products regulated by changes in the redox state of the cell: APX [62], glycolate oxidase [63], Rubisco activase [64], Rubisco [65], plastid glucose-6-phosphate dehydrogenase [66], and 3-deoxy-D arabino heptosonate-7 phosphate synthase [67]. It is likely that, in addition to regulating protein activity, the reductive power of the cell plays an important role in regulating the transcription of these genes, possibly through a feedback mechanism involving an interruption in the photosynthetic apparatus. We also observed that a number of genes were differentially expressed from basal levels in both treatments but at different time points. In the incompatible response, these genes were differentially regulated relative to basal level at 6 HPI while in the compatible response the transcriptional differences occurred at 12 HPI. The reason for this difference is unknown but it is tempting to speculate that the coordinated expression of pathways at 6 HPI rather than simply the induction or suppression of defence pathways at early stages of infection might determine the outcome of the pathogenic interaction. A microarray experiment examining gene expression differences between compatible and incompatible interactions of *P. syringae* and *A. thaliana* found that the expression of genes in the early hours of the incompatible infection resembled the expression of the same genes at much later stages in the compatible interaction [19]. They stated that this may indicate that incompatible and compatible interactions share signaling pathways but are induced at different times. To determine if this is the case between wheat and leaf rust as well, additional expression profiling experiments over a broader time span would need to be performed.

It is not known that the gene expression changes that were measured at the early stages of infection lead to the induction of HR. It could be that the induction did not occur until later in the infection process. The possibility exists that this study did not measure Thatcher *Lr1* undergoing compatible and incompatible reactions due to the presence or absence of the *avr Lr1* gene, but instead measured differential expression due to other genetic difference that exists between fungal races BBB and TJB. However, given that many of the genes identified in this study are differentially expressed during the HR and gene-for-gene recognition responses in other systems, it is likely that the start of the incompatible and compatible reactions due to *avr Lr1 Lr1* was being observed.

Taken together, this study allowed us to show that phenotypic differences between the incompatible and compatible interactions in the wheat near-isogenic line RL6003/*P. triticinia* pathosystem are also reflected by differential expression of mRNA transcripts that occur very early after infection. Enzymes involved in photosynthesis and in the scavenging of reactive oxygen species, signal transduction and ubiquitination, as well as those of the shikimate-phenylpropanoid pathway, are strongly implicated as key determinants in RL6003 metabolism against incompatible pathogenic fungi. These findings indicate that synergistic and conserved strategies are utilized by the incompatible wheat host to fight against *P. triticina*.

## Supplementary Material

Supplementary Table 1. Genes differentially expressed between treatments with virulent and avirulent races of *P. triticina*
The EST sequences that were found to be differentially expressed in this study at a *P* value < = .055 for the interaction type by time effect are listed in the table. For each sequence, the following information is provided: the GenBank accession, the putative function assigned to the sequence based on BLASTX hit homology, the E value of the BLASTX assigned annotation, the expression values for treatments MB and MT, respectively, at the four time points, and the ANOVA *P* values for pathogen type, time and pathogen type x time effects.
